# Induction of matrix metalloprotease-1 gene expression by retinoic acid in the human pancreatic tumour cell line Dan-G

**DOI:** 10.1038/sj.bjc.6690446

**Published:** 1999-06

**Authors:** Z von Marschall, E-O Riecken, S Rosewicz

**Affiliations:** Medizinische Klinik I, Gastroenterologie/Infektiologie, Klinikum Benjamin Franklin, Hindenburgdamm 30, Berlin, 12200, Germany

**Keywords:** matrix metalloprotease-1, retinoic acid, pancreas

## Abstract

We have investigated the effects of retinoic acid (RA) on matrix metalloprotease-1 (MMP-1) gene expression in the human pancreatic tumour cell line Dan-G. 13-*cis* RA results in a time- and dose-dependent increase of MMP-1 protein concentration. These stimulatory effects were paralleled by a time- and dose-dependent increase of MMP-1 mRNA steady-state concentrations. Nuclear run-on analysis revealed that the increase of MMP-1 mRNA was partially due to an increase of MMP-1 gene transcription. In addition, 13-*cis* RA treatment results in an increase of MMP-1 mRNA stability. These data demonstrate that RA stimulates MMP-1 gene expression in human pancreatic carcinoma cells by transcriptional and post-transcriptional mechanisms. © 1999 Cancer Research Campaign


*British Journal of Cancer* (1999) **80**(7), 935–939
© 1999 Cancer Research Campaign
Article no. bjoc.1998.0446

### Induction of matrix metalloprotease-1 gene expression by retinoic acid in the human pancreatic tumour cell line Dan-G

**Z von Marschall, E-O Riecken and S Rosewicz**

Medizinische Klinik I, Gastroenterologie/Infektiologie, Klinikum Benjamin Franklin, Hindenburgdamm 30, 12200 Berlin, Germany

**Summary** We have investigated the effects of retinoic acid (RA) on matrix metalloprotease-1 (MMP-1) gene expression in the human pancreatic tumour cell line Dan-G. 13-*cis* RA results in a time- and dose-dependent increase of MMP-1 protein concentration. These stimulatory effects were paralleled by a time- and dose-dependent increase of MMP-1 mRNA steady-state concentrations. Nuclear run-on analysis revealed that the increase of MMP-1 mRNA was partially due to an increase of MMP-1 gene transcription. In addition, 13-*cis* RA treatment results in an increase of MMP-1 mRNA stability. These data demonstrate that RA stimulates MMP-1 gene expression in human pancreatic carcinoma cells by transcriptional and post-transcriptional mechanisms.

**Keywords:** matrix metalloprotease-1; retinoic acid; pancreas

Matrix metalloprotease-1 (MMP-1) is a member of the large family of metalloproteases that play an important role in the remodelling of the extracellular matrix (Matrisian, 1990; Woessner, 1991). In addition to MMP-2, -8, -13 and -14, MMP-1 is capable of degrading the interstitial collagens I, II and III at a neutral pH (Nagase et al, 1983). Based on these properties, it has been suggested that MMP-1 plays a key role in the regulation of local tumour growth, invasion and metastasis (Salo et al, 1983; Matrisian, 1990). Representing a potential therapeutic target, various substances have been investigated, capable of inhibiting MMP gene expression. In this context, the effects of retinoic acid (RA) on MMP gene expression have been studied in a wide variety of different cells and tissues. A rather complex picture has emerged from these studies: while RA stimulates MMP-13 gene expression (which shows high homology to human MMP-1) in rat osteosarcoma cells and rat primary osteoblasts (Conolly et al, 1994; Varghese et al, 1994), inhibition of MMP-1 gene expression has been observed in synovial cells (Brinckerhoff et al, 1980), monocytes (Ohta et al, 1987), fibroblasts (Brinckerhoff and Harris, 1981) and epidermal keratinocytes (Bailly et al, 1990); these data therefore suggest that regulation of MMP gene expression by retinoic acid occurs in a cell type- and tissue-specific manner. It should be kept in mind that the interstitial collagenase in rat tissues (MMP-13) is highly homologous, but not identical to the human collagenase-3.

It has recently become increasingly clear that epithelial tumour cells themselves can also act as a source of MMP-1 synthesis and secretion, thereby directly modulating degradation of the surrounding extracellular matrix (Chen, 1992). Although RA treatment has been explored in a wide variety of human malignancies (Bollag and Holdener, 1992), very little is currently known about the regulation of MMP-1 gene expression by RA in human epithelial cancer cells. As an initial attempt to address this issue, in the current study we established the human pancreatic carcinoma cell line Dan-G as an in vitro model to investigate the effects of RA on MMP-1 gene expression in human pancreatic cancer cells.

*Received 23 July 1998*
*Revised 15 December 1998*
*Accepted 16 December 1998*

*Correspondence to:* S Rosewicz

### MATERIALS AND METHODS

### Cell culture

The human pancreatic carcinoma cell line Dan-G was obtained from the Deutsche Krebsforschungszentrum (Heidelberg, Germany). We have previously demonstrated by cytokeratin phenotyping, as well as expression of the duct cell-specific marker gene carbonic anhydrase II, that this cell line represents a ductal phenotype (Rosewicz et al, 1995*a*, 1995*b*). Dan-G cells were grown in DMEM supplemented with 10% fetal calf serum (FCS). Cells were kept under 95% air and 5% carbon dioxide at 37°C. 13-*cis* RA was added from stock solutions, prepared under subdued light. Control cells received ethanol as a vehicle control, and the final concentration of ethanol in the medium did not exceed 0.1%.

### Western blots

Cells were cultured in 10-cm Petri dishes in the presence of 10% FCS until confluency. Medium was then removed and the cells were thoroughly washed three times to remove serum. Cells were then switched to serum-free medium with or without the specified retinoid. After the indicated time intervals, the supernatant was collected and precipitated with 3% trichloracetic acid (TCA). Protein samples (10 μg per sample) were then electrophoresed on a 12% sodium dodecyl sulphate (SDS) polyacrylamide gel under reducing conditions. Proteins were then electroblotted onto nitrocellulose membranes. Non-specific binding was blocked by 5% (w/v) fat-free milk solution. Blots were then incubated with a rabbit polyclonal antibody directed against human MMP-1 (Quartett, Berlin, Germany; cat: 5980-0170) at a final dilution of 1:100

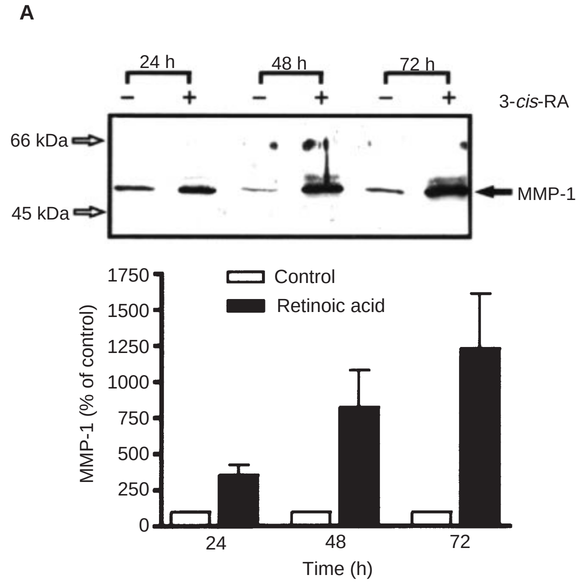

**Figure 1** Effects of 13-*cis* retinoic acid on MMP-1 expression in Dan-G cells. A total of 10 μg TCA-precipitated Dan-G cell supernatant protein were analysed by Western blotting using a monospecific polyclonal antibody against human MMP-1, as described in Methods. Shown is a representative Western blot (**A**) and the quantitative analysis of three independent experiments (**B**) (values are given as mean ± s.e.m.)

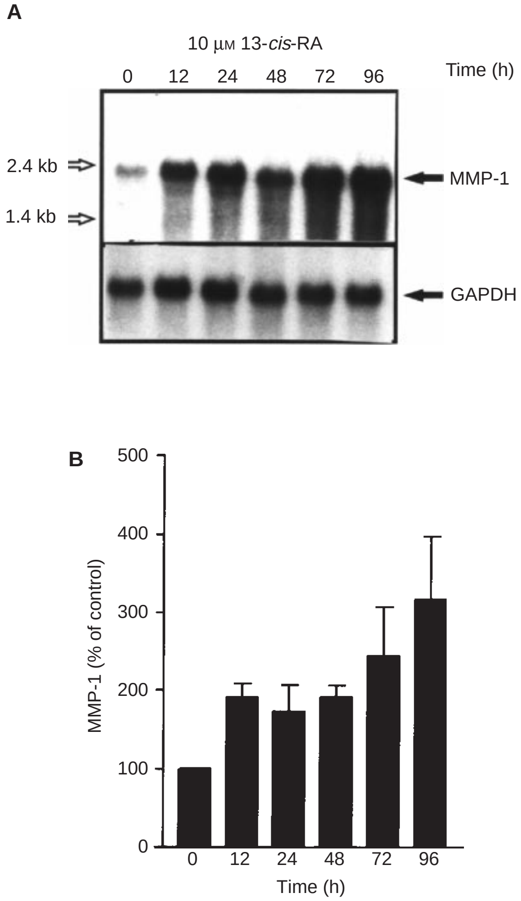

**Figure 2** Effects of 13-*cis* retinoic acid on MMP-1 mRNA concentrations. Dan-G cells were incubated for the indicated time points with 10 μM 13-*cis* RA. Then, 20 μg RNA were analysed by Northern blotting using random-primed cDNA probes against human MMP-1 and GAPDH, which served as an internal loading control. Shown is a representative Northern blot (**A**). The signals were then quantitated by densitometry and normalized to GAPDH. Shown are the mean ± s.e.m. of three independent experiments (**B**)

overnight. After extensive washing, blots were then reacted with goat anti-rabbit IgG conjugated to alkaline phosphatase as a second antibody. Protein bands were subsequently detected using alkaline phosphatase-dependent nitroblue tetrazolium–bromochloroindoyl phosphate reduction. The detected signals were then quantitated by laser densitometry. Identical results were obtained with a mouse monoclonal anti-human MMP-1 antibody (R&D Sytems, Wiesbaden, Germany; cat: MAB901).

### Northern blots

RNA was isolated by the guanidinium isothyanate procedure (Chirgwin et al, 1979). RNA was denatured by formaldehyde, subjected to electrophoresis through a 1% agarose gel in the presence of formaldehyde and then transferred onto nitrocellulose. Prehybridization, hybridization and washing procedures were carried out exactly as previously described (Rosewicz et al, 1994), using a random primed cDNA probe for human MMP-1. All filters were stripped and then sequentially hybridized with a cDNA-encoding human glyceraldehyde 3-phosphate dehydrogenase (GAPDH), which was used as a loading control. The hybridization signal was quantitated by laser densitometry, normalized to GAPDH and then expressed as % of control.

### Nuclear run on analysis

Nuclei were isolated by sucrose gradient centrifugation. RNA products were purified using deoxyribonuclease, proteinase K and salt precipitation according to the procedure of Nelson and Groudine (1986). Care was taken that each experimental condition contained the same amount of nuclei and radioactivity. Prehybridization, hybridization and washing procedures were carried out exactly as previously described (Rosewicz et al, 1994).

### mRNA stability studies

Dan-G cells were pretreated for 24 h with 13-*cis* RA or vehicle. RNA synthesis was then inhibited by the addition of 150 μM of 5,6-dichloro-1β-D-ribofuranosylbenzimidazole (DRB). RNA was isolated at the indicated time points. MMP-1 mRNA concentrations were then quantitated by slot-blot analysis exactly as previously described (Rosewicz et al, 1994).

### RESULTS

### 13-*cis* RA stimulates MMP-1 gene expression in human pancreatic Dan-G cells

Using a monospecific antibody against human MMP-1 in Western blotting of Dan-G cell supernatants, we detected a single band of 52 kDa corresponding to the secreted MMP-1 proenzyme (Figure 1A). To evaluate the effects of retinoids, we chose 13-*cis* RA at a final concentration of 10 μM because we have previously found that this concentration is maximally effective in terms of growth inhibition and induction of cellular differentiation in human pancreatic carcinoma cells (Rosewicz et al, 1995*a*). Incubation with 13-*cis* RA

*© 1999 Cancer Research Campaign*

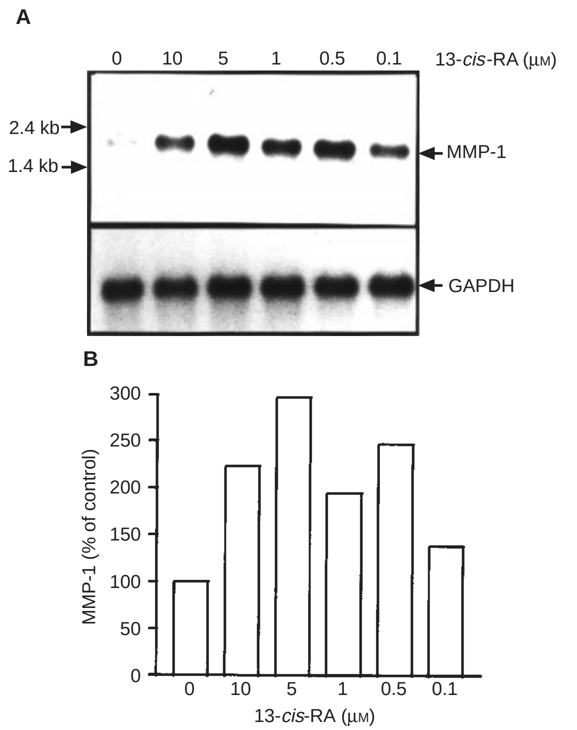

**Figure 3** Dose-dependent effects of 13-*cis* retinoic acid on MMP-1 gene expression. Dan-G cells were incubated for 24 h with the indicated concentrations of 13-*cis* RA. RNA was then extracted and analysed by Northern blotting (**A**). The signals were then quantitated by densitometry and normalized to GAPDH (**B**)

resulted in a time-dependent increase of MMP-1 concentrations compared to untreated controls, which was detectable as early as 24 h (357 ± 71% of control) and reached a maximum after 72 h of treatment (1234 ± 381% of control). To further characterize the underlying mechanisms responsible for MMP-1 stimulation by 13-*cis* RA, we next investigated the effects on MMP-1 mRNA concentrations. Using a cloned human cDNA for MMP-1 in Northern blotting we detected a single mRNA species of 2.3 kb corresponding to what has previously been described for human MMP-1 (Figure 2) (Goldberg et al, 1986). Treatment of Dan-G cells with 13-*cis* RA resulted in a time-dependent increase of MMP-1 mRNA concentrations, which was detectable as early as 12 h and reached maximal effects at 96 h, while GAPDH which served as an internal control did not change under any experimental condition (Figure 2). The effects of 13-*cis* RA on MMP-1 gene expression were dose-dependent with half-maximal effects observed at 100 nM and maximal stimulation occurring at concentrations of 5 µM (Figure 3). When we tested various naturally occurring retinoid analogues in respect to their potency to stimulate MMP-1 gene expression in Dan-G cells, we observed the following gradient based on equimolar concentrations (10 µM): 13-*cis* RA (234 ± 42% of control, $n = 3$) > all-*trans* RA (183 ± 24% of control, $n = 3$) > 9-*cis* RA (142 ± 2% of control, $n = 3$).

### Effects of 13-*cis* RA on MMP-1 mRNA stability

To further analyse the molecular mechanisms responsible for the observed induction of MMP-1 mRNA steady-state concentrations, we first investigated the effects of 13-*cis* RA on MMP-1 mRNA stability. Preincubation of Dan-G cells with 10 µM 13-*cis* RA for

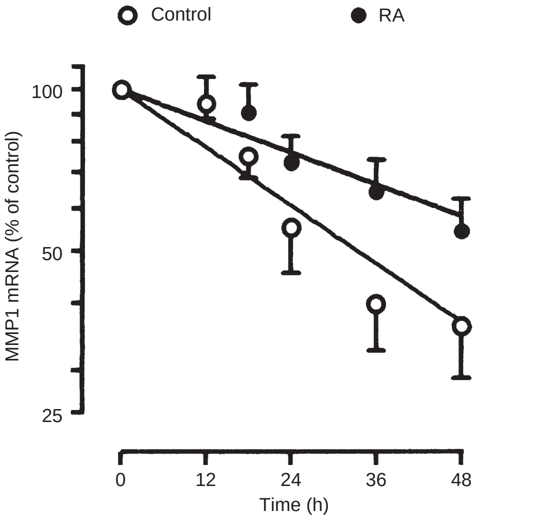

**Figure 4** 13-*cis* retinoic acid increases MMP-1 mRNA half-life. Dan-G cells were pretreated with 13-*cis* retinoic acid or vehicle for 24 h. RNA synthesis was then inhibited by the addition of 150 µM DRB. After the indicated time points. MMP-1 mRNA was analysed by slot-blot analysis and quantitated by laser densitometry. Values are given as mean ± s.e.m. of three independent experiments

24 h resulted in a considerable increase of MMP-1 mRNA stability. The MMP-1 mRNA half-life was calculated to be 31.1 h (95% confidence interval (CI) 26.2–38.1 h) in control cells in contrast to 57.8 h (95% CI 48.1–72.6 h) after treatment with 13-*cis* RA (Figure 4). Extended treatment beyond 48 h of Dan-G cells with DRB was not possible due to unacceptable cell toxicity as a consequence of prolonged inhibition of RNA synthesis.

### Effects of 13-*cis* RA on MMP-1 gene transcription

Although 13-*cis* RA significantly increased MMP-1 mRNA half-life, this mechanism cannot fully account for the extent of the observed increase in MMP-1 mRNA concentrations (e.g. MMP-1 mRNA concentrations were 192 ± 20% of controls after 12 h of 13-*cis* RA treatment) (Figure 2). We therefore examined whether 13-*cis* RA might in addition regulate MMP-1 gene transcription. Using the nuclear run on technique in control nuclei and nuclei that had been pretreated with 13-*cis* RA for 24 h we repeatedly detected an increased hybridization signal for MMP-1 in the RA pretreated cells (Figure 5). Densitometric analysis revealed a MMP-1 transcription rate of 185 ± 13% of controls ($n = 3$) after 13-*cis* RA pretreatment for 24 h. Retinoic acid did not increase transcription non-specifically because β-actin gene transcription was not altered under any experimental condition (Figure 5).

### DISCUSSION

In the current study, we investigated the effects of RA on MMP-1 gene expression in human pancreatic carcinoma cells. Using a mono-specific antibody as well as a human cDNA probe we were able to demonstrate MMP-1 protein and mRNA expression in the pancreatic carcinoma cell line Dan-G. These results are in good agreement with a recent study, demonstrating MMP-1 expression in two highly metastatic pancreatic carcinoma cell lines (Jimi et al, 1997). Furthermore, we think that this in vitro system is relevant to the in vivo biology of pancreatic cancer, because we have subsequently

*© 1999 Cancer Research Campaign* 

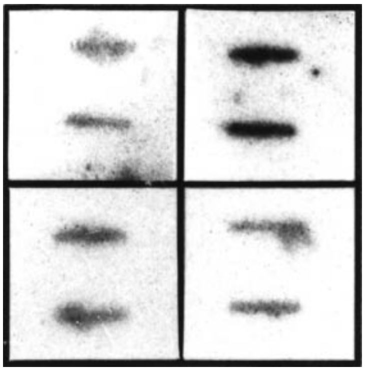

**Figure 5** Effects of 13-*cis* retinoic acid on MMP-1 gene transcription in Dan-G cells. Dan-G cells were incubated with 10 μM 13-*cis* RA for 24 h and MMP-1 and β-actin transcription were assessed by nuclear run on assays. Duplicate slots for each cDNA were hybridized to ensure the reproducibility. A representative of three independent experiments is shown found by immunohistochemistry, that ~50% of all human pancreatic tumours express MMP-1 protein in the ductal cancer cells, whereas non-transformed ductal cells are negative, suggesting a central role of MMP-1 in the remodelling process of the tumour-associated extracellular matrix of pancreatic cancer (von Marschall and Rosewicz, manuscript in preparation).

RA has been shown to regulate MMP-1 gene expression in a cell type- and tissue-specific manner (Brinckerhoff et al, 1981, 1980; Ohta et al, 1987; Bailly et al, 1990; Conolly et al, 1994; Varghese et al, 1994). Depending on the cell system investigated, induction as well as inhibition of MMP gene expression by RA have been described, although these regulatory processes are currently poorly understood in epithelial cancer cells. Using Dan-G cells as an in vitro model we observed a time- and dose-dependent induction of MMP-1 protein concentrations by RA. The increase of MMP-1 protein was paralleled by an increase in MMP-1 mRNA concentrations, suggesting that RA action occurs mainly at a pretranslational level. The effects of retinoids are believed to be mediated by two families of nuclear receptors, designated retinoic acid receptors (RAR) and retinoid X receptors (RXR), each consisting of three receptor subtypes, named α, β and γ (Leid et al, 1992; Giguere, 1994). Both RAR and RXR act as ligand-activated transcription factors, controlling gene transcription initiated from promotors of retinoid-regulated genes by interacting with *cis*-acting elements, the so-called RAREs (retinoic acid responsive elements) (Leid et al, 1992; Giguere, 1994). We have previously shown by reverse transcription polymerase chain reaction (RT-PCR) that the human Dan-G cell line does express all RAR and RXR subtypes, except for the RXRγ (Rosewicz et al, 1995*a*). Although most of the biological effects of retinoids are believed to occur via transcriptional gene regulation, modulation of mRNA stability by RA has been previously described (Zhou et al, 1994). To further dissect the molecular mechanisms responsible for RA-mediated MMP-1 induction, we performed mRNA half-life studies and nuclear run-on assays in Dan-G cells. As a result of these experiments, we observed a dual mode of RA action: stimulation of MMP-1 gene transcription and an increase of MMP-1 mRNA stability; both mechanisms contribute to the observed increase of MMP-1 mRNA and protein concentrations.

This combined regulatory control of MMP-1 gene expression represents a novel finding compared to what has been previously described for RA-mediated MMP-1 expression in other cell systems: in mesenchymal and blood cells, RA results in transcriptional inhibition of MMP-1 without affecting MMP-1 mRNA stability (Brinckerhoff et al, 1980; Brinckerhoff and Harris, 1981; Ohta et al, 1987); this inhibition is believed to be due to protein–protein interactions between the retinoid receptors (RARs/RXRs) and the AP-1 transcription factor rather than direct binding of retinoid receptors to regulatory sequences of the MMP-1 gene (Schule et al, 1991; Pan et al, 1995). In contrast to the regulatory effects of RA, Dan-G cells react in a similar fashion to mesenchymal or blood cells in response to other extracellular modulators of MMP-1 gene expression. For example, treatment with the phorbolester TPA results in a time-dependent stimulation of MMP-1 gene expression in Dan-G cells (data not shown).

In contrast to the regulation of MMP-1 in Dan-G cells, RA stimulates MMP-13 gene transcription in rat osteosarcoma cells (Conolly et al, 1994), whereas it increases MMP-13 mRNA half-life in rat primary osteoblasts (Varghese et al, 1994). Taken together, these data suggest that the composition of the cell type-specific transcription machinery critically determines the mode of RA-mediated regulation of MMP gene expression.

In summary, the current study provides the first evidence of combined transcriptional and post-transcriptional stimulation of MMP-1 gene expression by RA in a newly established in vitro model of a MMP-1 expression human pancreatic carcinoma cell line.

### ACKNOWLEDGEMENTS

This work was supported by a grant from the Mildred Scheel Stiftung (10-1265) to SR.

### REFERENCES

Bailly C, Dreze S, Asselineau D and Nusgens B (1990) Retinoic acid inhibits the production of collagenase by human epidermal keratinocytes. *J Invest Dermatol* **94**: 47–51

Bollag W and Holdener EE (1992) Retinoids in cancer prevention and therapy. *Ann Oncol* **3**: 512–526

Brinckerhoff C, McMillan RM, Dayer JM and Harris ED (1980) Inhibition by retinoic acid of collagenase production in rheumatoid synovial cells. *N Engl J Med* **303**: 332–336

Brinckerhoff C and Harris ED (1981) Modulation by retinoic acid and corticosteroids of collagenase production by rabbit synovial fibroblasts treated with phorbol myristate acetate or polyethylene glycol. *Biochim Biophys Acta* **677**: 424–432

Chen WT (1992) Membrane proteases: roles in tissue remodeling and tumor invasion. *Curr Opin Cell Biol* **4**: 802–809

Chirgwin JM, Przybyla AE, MacDonald RJ and Rutter WJ (1979) Isolation of biologically active ribonucleic acid from sources enriched in ribonuclease. *Biochemistry* **18**: 5294–5299

Conolly TJ, Clohishy JC, Shilt JS, Bergman KD, Partridge NC and Quinn CO (1994) Retinoic acid stimulates interstitial collagenase messenger ribonucleic acid in osteosarcoma cells. *Endocrinology* **135**: 2542–2548

Giguere V (1994) Retinoic acid receptors and cellular retinoid binding proteins: complex interplay in retinoid signalling. *Endocrine Rev* **15**: 61–79

Goldberg GI, Wilhelm SM, Kronberger A, Bauer EA, Grant GA and Eisen AZ (1986) Human fibroblast collagenase. *J Biol Chem* **261**: 6725–6729

Jimi SI, Shono T, Ono M, Kuwano M, Tanaka M, Lopez-Otin C and Kono A (1997) Expression of matrix metalloproteinases 1 and 2 genes in a possible association with metastatic abilities of human pancreatic cancer cells. *Int J Oncol* **10**: 623–628

Leid M, Kastner P and Chambon P (1992) Multiplicity generates diversity in the retinoic acid signalling pathways. *Trends Biol Sci* **17**: 427–433

Matrisian LM (1990) Metalloproteinases and their inhibitors in matrix remodeling. *Trends Genet* **6**: 121–125

© 1999 Cancer Research Campaign

Nagase H, Brinckerhoff C, Vater CA and Harris ED (1983) Biosynthesis and secretion of procollagenase by rabbit synovial fibroblasts. *Biochem J* **214**: 281–288

Nelson JA and Groudine M (1986) Transcriptional regulation as assayed by an in vitro elongation system. *Mol Cell Biol* **6**: 452–461

Ohta A, Louis JC and Uitto J (1987) Retinoid modulation of collagenase production by adherent mononuclear cells in culture. *Ann Rheum Dis* **46**: 357–362

Pan L, Eckhoff C and Brinckerhoff CE (1995) Suppression of collagenase gene expression by all-trans and 9-cis retinoic acid is ligand dependent and requires both RARs and RXRs. *J Cell Biochem* **57**: 575–589

Rosewicz S, Detjen K, Kaiser A, Prosenc N, Cervos-Navarro J, Riecken EO and Haller H (1994) Bombesin receptor gene expression in rat pancreatic acinar AR42J cells: transcriptional regulation by glucocorticoids. *Gastroenterology* **107**: 208–221

Rosewicz S, Stier U, Brembeck F, Kaiser A, Papadimitriou C, Berdel WE, Wiedenmann B and Riecken EO (1995*a*) Retinoids: effects on growth, differentiation, and nuclear receptor expression in human pancreatic carcinoma cell lines. *Gastroenterology* **109**: 1646–1660

Rosewicz S, Riecken EO and Stier U (1995*b*) Transcriptional regulation of carbonic anhydrase II by retinoic acid in the human pancreatic tumor cell line DanG. *FEBS Lett* **368**: 45–48

Salo T, Liotta L and Tryggvason K (1983) Purification and characterization of a murine basement membrane collagen-degrading enzyme secreted by metastatic tumor cells. *J Biol Chem* **258**: 3058–3063

Schüle R, Rangarajan P, Kliewer S, Ansone LJ, Bolado J, Verma IM and Evans RM (1991) Retinoic acid is a negative regulator of AP-1 responsive genes. *Proc Natl Acad Sci USA* **88**: 6092–6096

Varghese S, Rydziel S, Jeffrey JJ and Canalis E (1994) Regulation of interstitial collagenase expression and collagen degradation by retinoic acid in bone cells. *Endocrinology* **134**: 2438–2444

Woessner JJ (1991) Matrix metalloproteinases and their inhibitors in connective tissue remodeling. *FASEB J* **5**: 2145–2154

Zhou H, Manjii SS, Findla DM, Martin JH, Heath JK and Ng KW (1994) Novel action of retinoic acid. *J Biol Chem* **269**: 22433–22439

*© 1999 Cancer Research Campaign*
